# Prediction of metastasis-free survival in patients with localized prostate adenocarcinoma using primary tumor and lymph node radiomics from pre-treatment PSMA-PET/CT scans

**DOI:** 10.1016/j.radonc.2025.111119

**Published:** 2025-09-06

**Authors:** Apurva Singh, William Silva Mendes, Sang-Bo Oh, Ozan Cem Guler, Aysenur Elmali, Birhan Demirhan, Amit Sawant, Phuoc Tran, Cem Onal, Lei Ren

**Affiliations:** a Department of Radiation Oncology, University of Maryland School of Medicine, Baltimore, MD, USA; b Baskent University Faculty of Medicine, Adana Dr Turgut Noyan Research and Treatment Center, Department of Radiation Oncology, Adana, Turkey; c Baskent University Faculty of Medicine, Department of Radiation Oncology, Ankara, Turkey; d Division of Medical Oncology and Hematology, Department of Internal Medicine, Pusan National University Yangsan Hospital, Pusan National University School of Medicine, Yangsan, Republic of Korea

## Abstract

**Purpose:**

To predict metastasis-free survival (MFS) for patients with prostate adenocarcinoma (PCa) treated with androgen deprivation therapy (ADT) and external radiotherapy using clinical factors and radiomics extracted from primary tumor and node volumes in pre-treatment PSMA PET/CT scans.

**Materials/Methods:**

Our cohort includes 134 PCa patients (nodal involvement in 28 patients). Gross tumor volumes of primary tumor (GTVp) and nodes (GTVn) on CT and PET scans were segmented. A 5 mm expansion ring area outside primary tumor was defined. Z-score normalization was applied to radiomics features extracted from tumor and ring; dimension reduction was performed using Principal Components Analysis (PCA). For patients with only primary tumor, we took 3 principal components (PCs) from GTVp and one ring PC as representative radiomics components from CT and PET scans. For patients with nodes, we calculated weighted average (by volume) of radiomics from primary tumor and nodes, computed first 3 PCs and combined it with 1st PC from the ring. Radiomics PCs and clinical variables (age, Gleason score, initial prostate specific antigen value (i PSA), PSA_relapse) formed the predictors. Due to MFS data imbalance (metastasis-24, no metastasis-110), we performed 70:30 train-test split and applied imbalance correction to training data. Univariate Cox-regression was used to select top predictors (logistic regression p < 0.05). Multivariate Cox-regression was performed on imbalance-corrected training data and fit on testing data (using predictors selected from training). Model 2 was built using clinical variables and radiomic PCs from primary tumors (GTVp, ring). Model 3 was built using clinical variables only. Binary classification analysis for prediction of five-year MFS was also performed.

**Results:**

Results of time-to-event analysis (MFS) were: Cox-regression c-scores: model1: train- 0.77 [0.72, 0.78]; test- 0.69 [0.64, 0.70]; model2: train- 0.72 [0.66, 0.73]; test- 0.63 [0.58, 0.64]; model3: train- 0.62 [0.57, 0.63]; test- 0.54 [0.51, 0.56]. The results of 5 year MFS classification analysis were [sensitivity, specificity, AUC]: model 1: train- [83.6 %, 91.3 %, 0.88]; test- [76.3 %, 82.5 %, 0.81]; model 2: train- [77.4 %, 85.1 %, 0.84]; test- [71.5 %, 78.2 %, 0.76]; model 3: train- [69.3 %, 78.2 %, 0.76]; test- [64.7 %, 72.6 %, 0.68]. The two cohorts of patients classified by model 1 showed statistically significant differences in their actual survival curves, demonstrating the efficacy of the classification. Integration of node with primary tumor-radiomics provides the best prognostic performance in MFS prediction.

**Conclusion:**

This is one of the first studies to explore the prognostic value of pre-treatment PSMA-PET, a relatively recent advancement in the care of prostate adenocarcinoma patients. Results demonstrated the potential of using imaging biomarkers from PSMA-PET/CT images for prognosis prediction before the treatment, which provides clinicians valuable information for customizing the treatment paradigm to improve the outcomes for primary prostate cancer patients.

## Introduction

Prostate cancer is the second most common cancer and is one of the leading causes of cancer-related deaths in men across the world [1]. Assessment by prostate specific antigen (PSA) levels is the primary means for the screening of prostate cancer [2,3]. Patients diagnosed with primary prostate cancer have surgery and radiation as the two treatment options [4]. Intensity-modulated radiation therapy (IMRT), that can deliver a precise dose to the prostate and reduce risk to the surrounding organs, is the dominant radiation modality [5]. Brachytherapy and stereotactic body radiation therapy (SBRT)/ stereotactic ablative radiation therapy (SABR) are other common radiation methods [6,7]. In higher risk patients receiving radiation therapy, concurrent administration of androgen deprivation therapy (ADT) is also recommended in the course of treatment [8]. Despite the progress, prostate cancer recurrence after primary therapy has been observed in 20–60 % of the cases [9,10]. For patients with high volume metastatic disease, the five-year survival rate is below 30 % [11]. Therefore, there is an urgent need to develop effective prognostic biomarkers to predict patient responses from therapy so that treatments can be customized to improve the outcomes.

Prostate specific membrane antigen positron emission tomography (PSMA-PET) is a promising functional imaging modality used for initial diagnosis and treatment planning of prostate cancer [12,13]. PSMA is a transmembrane glycoprotein expressed on the surface of prostate cancer cells, and has several important functions including angiogenesis, cell migration and nutrition [14]. Since PSMA is overexpressed by local and metastatic prostate cancer cells, the overexpression of PSMA is used as an important molecular biomarker in prostate cancer. Molecular imaging has shown excellent sensitivity and specificity in assessing patients with presumed metastatic prostate cancer [12,15,16]. PSMA-PET imaging has been shown to improve the accuracy of staging by detecting primary tumor and node metastases, while ruling out distant metastases [12,17–21]. PSMA-PET-based imaging can also provide prognostic values for the non-invasive identification of patients with better response from radiation therapy before the treatment, which is critical for the customization of treatment paradigm to improve outcomes.

Radiomic analysis has emerged as a powerful tool for extracting quantitative imaging features that serve as biomarkers for prognostic prediction in cancer patients [18–21]. Radiomics features, calculated based on the intensity, shape, size, volume and texture from the ROIs, can provide an in-depth in vivo analysis of the heterogeneity of the tumor and surrounding regions [22,23]. In comparison, standard biopsy procedures can be subject to sampling error, cause patient discomfort, pose a significant financial cost to the hospital [24,25]and thus are typically limited to be used at the time of initial diagnosis and for documenting recurrence. Radiomic biomarkers derived from patient cohorts involving computed tomography (CT) and PET scans have been analyzed and used to build prognostic biomarkers for the prediction of response to therapy [26,27]. The combination of information from PET and CT scans can provide a description of the structural and functional characteristics of the tumor and peritumoral regions. Developing radiomics biomarkers from PSMA-PET/CT scans can be valuable for prognostic prediction to guide decision making for patients with prostate cancer.

Previously, studies involving PSMA-PET scans for patients with prostate cancer have explored the prognostic value of change in the pre- and post-SABR PSMA-PET standardized uptake volume (SUV) as a biomarker for treatment response [28]. For instance, change in the PSMA-PET maximum standardized uptake volume (SUV_max_) over time was found to be an important biomarker that correlated with two-year metastasis-free survival (MFS) for oligometastatic prostate cancer patients [28,29]. The value of PSMA-PET radiomics as a biomarker in the prognosis of patients with prostate cancer has only been explored quite recently. In a study involving oligometastatic prostate cancer patients, Cao et al. investigated the use of radiomics biomarkers derived from pre- and post-metastasis-directed therapy (MDT) PSMA-PET images in the prediction of two-year MFS for oligometastatic prostate cancer patients treated by metastatic directed therapy [30]. However, there is a dearth of data on PSMA-PET-based radiomics biomarkers for prognosis prediction in the localized primary prostate cancer setting.

In our study, we have addressed this gap by accruing a cohort of 134 patients with prostate adenocarcinoma (primary tumors present in all cases, with 28 patients having nodal involvement). We have pre-registered PSMA-PET/CT images, giving us the advantage of multi-modal imaging information. We developed a novel scheme to integrate radiomics information from both primary tumors and pelvic nodes and combine them with initial-prostate-specific-antigen (iPSA), PSA-relapse, Gleason score and age data to build models for MFS prediction. We chose MFS as an endpoint in our study as it is a validated intermediate surrogate marker for overall survival in localized prostate cancer [31]. Imaging biomarkers have been successfully identified from the pre-treatment PSMA-PET/CT scans. To our knowledge, this is the first study to investigate radiomics biomarkers from pre-treatment PSMA-PET/CT scans for prognosis prediction for localized primary prostate cancer patients treated by radiation therapy, which paves the way for personalized treatment strategies, that could significantly enhance patient outcomes.

## Materials and methods

### Patient cohort

A total of 134 patients with prostate adenocarcinoma treated at Baskent University were retrospectively enrolled in the study with Institutional Review Board (IRB) approval (IRB number- HP-00100523). All methods in the study were in accordance with the Declaration of Helsinki under a waiver of informed consent. The inclusion criteria were: (i) confirmed histological diagnosis of prostate adenocarcinoma, (ii) clinically pelvic node-positive or node-negative disease based on PSMA-PET/CT evaluation, and (iii) treatment with ADT and external beam radiotherapy (EBRT). The exclusion criteria included: (i) prior radical prostatectomy, (ii) receipt of chemotherapy before radiotherapy, (iii) prostate-only radiotherapy, and (iv) documented distant metastases.

All patients received either 76–78 Gy or a focal boost of 86 Gy to the intraprostatic lesion using the simultaneous integrated boost technique. The radiation dose for the pelvic lymphatic field ranged from 46 to 54 Gy, without any additional boost to gross lymph nodes. ADT was prescribed to all patients, with the formulation and combination of drugs individualized at the discretion of the treating physician and in accordance with patient preference. ADT administration—including the decision to initiate therapy and the selection of agents—was based on the clinical judgment of the treating physician and patient preference. Androgen deprivation therapy consisted of a luteinizing hormone-releasing hormone (LHRH) agonist, either alone or in combination with first-generation antiandrogen bicalutamide. Further, next-generation androgen receptor pathway inhibitors (ARPIs), such as enzalutamide, abiraterone acetate, darolutamide, or apalutamide, were not administered. Likewise, no patients received upfront chemotherapy. This treatment approach reflects institutional standards during the study period (2017–2021), when ADT combined with radiotherapy was a widely accepted, guideline-concordant treatment for high-risk localized or node-positive prostate cancer. At that time, the routine use of ARPIs in non-metastatic high-risk settings had not yet become standard clinical practice. Moreover, access to ARPIs was restricted by national reimbursement regulations under the National Social Security System, which limited their use primarily to patients with metastatic castration-resistant prostate cancer. Therefore, the absence of ARPIs and chemotherapy in this cohort reflects both temporal treatment practices and healthcare system limitations. Likewise, poly (ADP-ribose) polymerase (PARP) inhibitors were not utilized in this cohort.

### Sample size consideration

Although this was a retrospective study, we performed a post hoc sample size and power estimation to assess the adequacy of our cohort size. Based on the Cox proportional hazards model, with 134 patients (24 experiencing metastasis and 110 without), the study provides approximately 82 % power to detect a hazard ratio (HR) of 1.8 in metastasis-free survival, assuming a two-sided alpha of 0.05 (brief description, including formulae for the above calculation has been included under the heading “Note 1: Sample size consideration- post hoc power analysis” in the supplemental file). This level of power is considered sufficient to detect moderate to strong associations between radiomic features and clinical outcomes. While prospective power calculations are preferable, this post hoc analysis supports the statistical validity of our sample size. Future prospective studies will incorporate formal sample size planning at the design stage.

### Overall workflow

A brief description of the workflow is included below:

1. *PET**/CT image pre-processing and segmentation-* PET and CT scan acquisition from Baskent University, segmentation of gross tumor volumes of primary prostate tumors (GTVp) and pelvic nodes (GTVn) on PET and CT scans, creation of a 5 mm ring expansion area outside GTVp, PET scans resampled to match voxel spacing of CT scans prior to radiomics feature extraction
2. *Clinical parameters and radiomics feature extraction*- selection of clinical parameters (age, Gleason score, initial prostate specific antigen value, PSA_relapse category) based on physician’s recommendation, extraction of radiomics features from GTVp, GTVn and ring regions using 3D Slicer PyRadiomics
3. *Feature normalization and dimensionality reduction*- Z-score normalization applied to the radiomics features and dimensionality reduction performed using principal components analysis (PCA)
4. *Model description-* For patients with primary prostate tumors only, we took three principal components (PCs) from GTVp and one ring region PC. For patients with nodes, we calculated the weighted average by volume of radiomics from the primary tumor and nodes, computed the first three PCs and combined with the first PC from the ring region. Model 1 was built using clinical variables and radiomics PCs from the weighted average of GTVp and GTVn and the radiomics PC from the ring region. Model 2 was built using clinical variables and radiomics PCs from primary tumors (GTVp, ring). Model 3 was built using clinical variables only.
5. Model training
  *5a.* *Time-to-event metastasis-free survival analysis- model training*- Due to MFS data imbalance (metastasis- 24, no metastasis- 110), we performed 70:30 train-test split and applied imbalance correction to train data. Univariate Cox regression was used to select top predictors. Multivariate Cox regression was performed on imbalance-corrected train data and fit on the test data.
  *5b.* *Five-year metastasis-free survival binary classification analysis- model training-* Binary classification analysis for prediction of five-year MFS was performed by selecting top predictors from the train data and using them to classify the patients with five-fold cross-validation in the test data.
6. Model testing and evaluation
  *6a.* *Time-to-event metastasis-free survival analysis- model evaluation-* The discrimination capacity of the models was assessed using the concordance statistic metric (c-score).
  *6b.* *Five-year metastasis-free survival binary classification analysis- model evaluation-* The evaluation of performance was done using the following metrics: sensitivity, specificity, area under the curve (AUC) and Kaplan-Meier curve comparison.

**Fig. 1.**
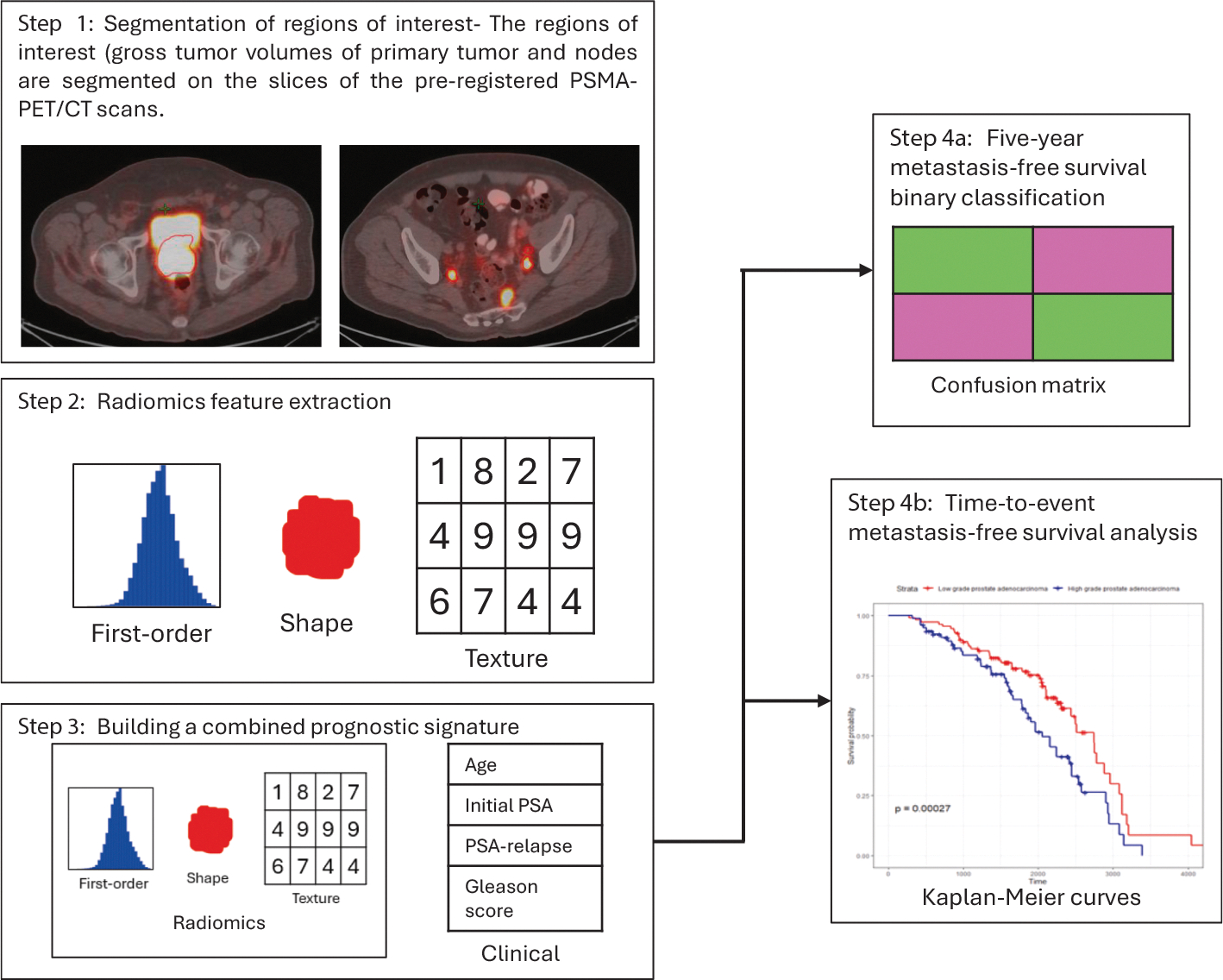
The workflow involving segmentation of regions of interest, radiomics feature extraction, combining radiomics and clinical information into a prognostic signature and performing time-to-survival event and binary classification analysis of metastasis-free survival.

This workflow has been represented below in Fig. 1.

Below is a more detailed description of each analysis step:

#### 1. PET/CT image pre-processing and segmentation

Patients were imaged at Başkent University using a dedicated PET/CT system (Discovery STE 8; GE Healthcare, Milwaukee, WI, USA). One section of the cohort had PET scans with original volumetric dimensions of 192*192*567 and voxel spacing of 3.65*3.65*3.27 mm^3^ and the other section of the cohort had PET scans with 128*128*367 and voxel spacing of 5.47*5.47*3.27 mm^3^. CT images were acquired with the original volumetric dimensions of 512*512*567 and voxel spacing of 1.37*1.37*3.26 mm^3^ and 140 kVp.

**Fig. 2.**
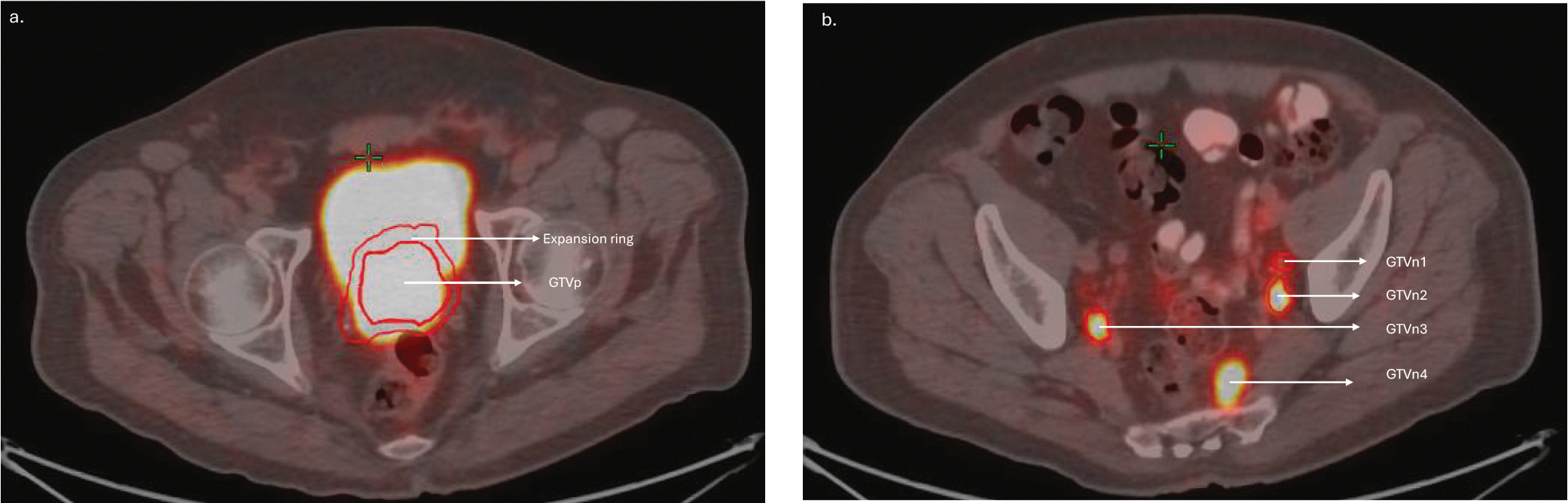
Representative figures showing the regions of interest including (a) the primary prostate tumor volume (GTVp) and expansion ring region and (b) the node regions (GTVn1–n4) on the pre-registered PSMA-PET/CT scan slices of a patient.

Gross tumor volume (GTV) of primary tumor (GTVp) and nodes (GTVn) based on PET and CT were identified and segmented by two radiation oncologists. A 5 mm expansion ring area outside the primary tumor GTV was created. The PET images were resampled to match the voxel spacing of the CT images prior to radiomics feature extraction. Fig. 2 below shows the ROIs on sample PET/CT scan slices of a patient.

#### 2. Clinical parameters and radiomics feature extraction

Clinical parameters including age, Gleason score, initial prostate specific antigen (i PSA) value and a categorical variable indicating PSA relapse, defined according to the Phoenix criteria, which describes biochemical failure as a rise of ≥2 ng/mL above the nadir PSA following radiotherapy [32]. The clinical and demographic information of the patients has been included in supplementary Table S1. The extraction of radiomics features was conducted using the 3D Slicer PyRadiomics [33,34]. A total of 107 radiomics features were extracted from each of the following regions in CT and PET images: gross tumor volumes of the GTVp, GTVn and ring regions. The radiomics features can be described under the following eight feature families: (i) Shape features- These features involve the description of the geometric aspects of the regions of interest, including area and volume. (ii) First-order features- These features describe the voxel gray-level intensities with the regions of interest. (iii) Gray level co-occurrence matrix features (GLCM)- These features describe the relationship between the neighboring pixels, such as correlations between the pixels and differences in intensity levels. (iv) Gray level dependence matrix features (GLDM)- These features quantify the gray level dependencies within the regions of interest (gray level dependency is defined as the number of connected voxels within a certain radius that are dependent on the center voxel). (v) Gray level run length matrix features- These features quantify the lengths of consecutive pixels as gray level runs. (vi) Gray level size zone matrix features- These features count the number of zones of linked voxels (voxels are said to be linked if the neighboring voxel has an identical discretized gray level). (vii) Neighboring gray tone difference matrix features- These features capture the coarseness of the texture of the regions of interest and are based on the gray-level relationships between the neighboring voxels. A list of features belonging to each feature family can be found in the supplementary Table S2.

#### 3. Feature normalization and dimensionality reduction

Z-score normalization was applied to the radiomics features extracted from the primary tumor, nodes and ring regions [35]. Dimensionality reduction was performed using Principal Components Analysis (PCA) [36].

#### 4. Model description

For patients with only primary prostate tumors, we took the first three PCs from the radiomics features extracted from GTVp and one principal component from the radiomics features extracted from the ring region as the representative radiomics components from CT and PET scans. For patients with nodes, we calculated the weighted average (by volume) of radiomics features extracted from GTVp and GTVn in the following manner:

$$\begin{array}{l} {}[\left[Rf(GTVp{)}^{*}v(GTVp)]+[Rf(GTVn1{)}^{*}v(GTVn1)\right]\\ \;+[Rf(GTVn2{)}^{*}v(GTVn2)]]/[v(GTVp)+v(GTVn1)+(v(GTVn2)] \end{array}$$

(Rf- radiomics feature, v- volume)

This is a representation of the computation of weighted average (by volume) of the radiomics features for a patient with two nodes in addition to the primary tumor.

The weighted average by volume technique allows for a more accurate integration of radiomics information from the primary tumor and other pelvic nodes by accounting for the varying contributions of each region of interest. This approach contrasts to simpler methods such as simply taking an average of the radiomics information derived from all the regions of interest.

We then computed the first three PCs from the weighted average radiomics features and combined them with the first PC from the radiomics features extracted from the ring region to form the representative radiomics components from the CT and PET scans. Radiomics PCs and clinical variables (age, Gleason score, iPSA, PSA_relapse) formed the list of predictors.

Model 1 was built using clinical variables and radiomics PCs from the weighted average of primary tumors (GTVp) and nodes (GTVn) and the radiomics PC from the ring region. Model 2 was built using clinical variables and radiomics PCs from the primary tumors (GTVp and ring regions). Model 3 was built using clinical variables only.

#### 5. Model training

##### 5a. Time-to-event metastasis-free survival analysis- model training.

Due to data imbalance in the metastasis event data (metastasis-24, no metastasis-110), we performed a 70:30 train-test split and applied imbalance correction to the train data using the Synthetic Minority Over-Sampling Technique (SMOTE) [37]. Univariate Cox-regression was used to select top predictors (logistic regression p < 0.05) from the imbalance-corrected train data [38]. Multivariate Cox-regression was performed on the imbalance-corrected train data and fit on the test data (using predictors selected from the train data) [39]. The data imbalance correction and Cox-regression analysis described above was performed using Python (Ver. 3.7, Anaconda) [40].

##### 5b. Five-year metastasis-free survival binary classification analysis- model training.

Binary classification analysis for prediction of five-year MFS was also performed by comparing the performance of various machine learning models in the Classification Learner App in MATLAB and selecting the classifier with the best performance [41]. The top predictors were selected from the train data (using the technique described above) and used to classify the patients based on five-year MFS in the test data with five-fold cross-validation.

#### 6. Model testing and evaluation

##### 6a. Time-to-event metastasis-free survival analysis- model evaluation

The discrimination capacity of the models was assessed using the concordance statistic metric (c-score) [42].

##### 6b. Five-year metastasis-free survival binary classification analysis- model evaluation

The evaluation of performance was done using the following quantitative metrics: sensitivity, specificity, area under the curve (AUC) and Kaplan-Meier curve comparison. The above models were used to classify patients into two groups based on five-year MFS. The Kaplan-Meier (KM) curves of the two groups were plotted using their actual survival data and compared to determine whether there is statistically significant difference in survival between the two groups classified by the model. We performed the KM plots using R programming language [43].

## Results

The results from the Cox-regression analysis for models 1–3 defined in the materials and methods section have been included below in Table 1.

We observed that the model built using radiomics information from primary prostate tumors, nodes, and ring structure and clinical variables (model 1) achieved the highest c-scores. Addition of radiomics information from the node regions in model 1 improves the prognostic performance compared to model 2 built using radiomics information only from the primary prostate tumor. Models 1 and 2 both have higher c-scores compared to the model built using only clinical variables. This indicates that addition of radiomics information to the clinical variables results in an improvement in the prognosis of metastasis-free survival.

**Fig. 3.**
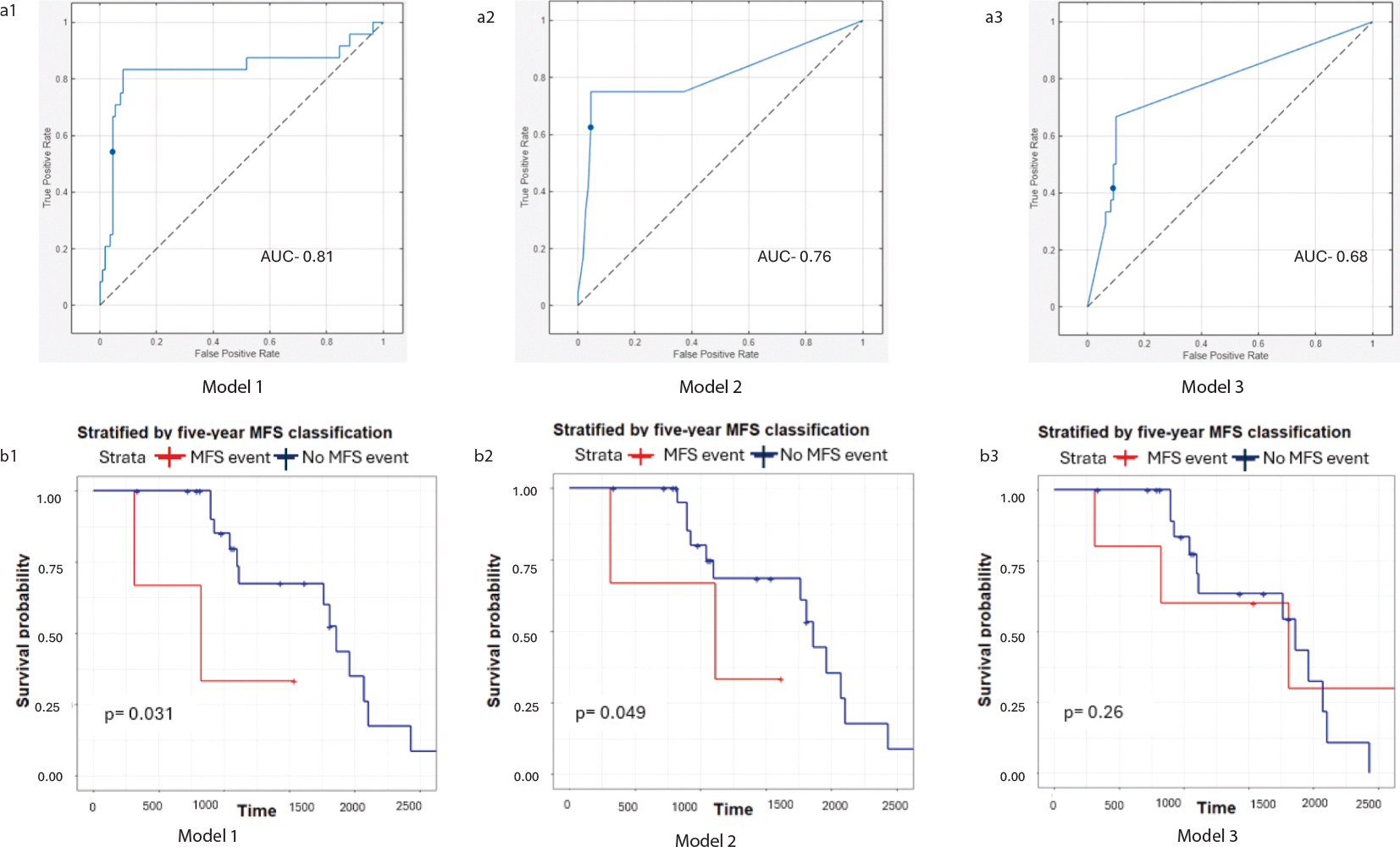
ROC curves (a1–a3) and KM survival curves (b1–b3) stratified by five-year metastasis-free survival classification results (MFS event vs no event) for models 1, 2 and 3.

The results from the five-year MFS classification analysis for models 1–3 defined in the materials and methods section have been included below in Table 2. For all three models, Quadratic SVM was the top performing classifier in the Classification Learner App in MATLAB and the results in Table 2 have been obtained using Quadratic SVM classifier. The AUC curves for the three models are shown in Fig. 3(a1–a3).

We observed that the model built using radiomics information from primary prostate tumors and nodes with clinical variables (model 1) achieved the highest classification accuracy. Addition of radiomics information from the node regions improves the prognostic performance compared to the model built using radiomics information only from the primary tumor. Models 1 and 2 both have higher classification accuracies compared to the model 3 built using only clinical variables. This indicates that the addition of radiomics information to the clinical variables results in an improvement in the five-year binary classification of metastasis-free survival.

The Kaplan-Meier metastasis free survival curves of patients stratified by five-year MFS classification results (event vs no event) have been included in Fig. 3 (b1–b3). We observed that the model built using radiomics information from primary prostate tumors and nodes with clinical variables (model 1) resulted in a statistically significant separation in the KM curves between patients classified as having MFS event vs no event (test data log rank p value: 0.031). The model built using clinical variables and radiomics information only from the primary prostate tumor resulted in a separation approaching statistical significance in the test data (log rank p value: 0.049). The model built using only clinical variables did not result in a statistically significant separation in KM curves in the test data (log rank p value: 0.26).

The results assessing the improvement in the Cox-regression analysis and five-year metastasis-free survival classification analysis results upon the addition of ring radiomics information to models 1 and 2 have been included in Table 3.

We observe that the addition of radiomics information from the ring expansion area improved the performance of our prognostic models slightly for both models 1 and 2.

## Discussion

PSMA-PET is a relatively new imaging technique that shows great potential for diagnosis and treatment assessment in prostate cancer patients. Existing studies in the literature involving PSMA-PET scans for patients with prostate cancer have explored the significance of change in the PSMA-PET SUV_max_ over time as an important biomarker that can be correlated with metastasis-free survival for oligometastatic prostate cancer patients [28,29]. The value of PSMA-PET radiomics as a prognostic biomarker for prognosis prediction in prostate cancer has only been recently explored in oligometastatic prostate cancer patients [30]. However, the investigation of PSMA-PET based prognostic radiomics biomarkers for primary prostate cancer patients is still a relatively unexplored area for this new imaging technique. The study by Moazemi et al. used a LASSO technique to identify relevant radiomics features from primary tumor and metastatic regions on pre-treatment PSMA-PET/CT scans and combined them with clinical parameters to predict overall survival in 83 patients with prostate cancer [44]. The study by Zang et al. also used a LASSO technique to develop a radiomics signature to differentiate between patients with prostate cancer from those with benign prostate disease [45].

In our study, we have accrued a cohort of 134 patients with prostate adenocarcinoma (primary prostate tumors present in all cases, with 28 patients having pelvic nodal involvement). We have developed imaging biomarkers using the radiomics information extracted from the gross tumor volumes of the primary prostate tumor, pelvic nodes and a ring expansion area around the primary prostate tumor, based on the pre-treatment PSMA-PET and CT scans. We have integrated the radiomics biomarker with clinical variables (iPSA), PSA-relapse, Gleason score and age) to enhance precision in the prediction of metastasis-free survival. To our knowledge, this is the first study to analyze prognostic radiomics biomarkers developed by computing the first principal component from the weighted average by volume of the radiomics features derived from the primary tumor and pelvic nodes. The weighted average technique presents a unique way to combine information from the various regions of interest and computing the principal components from the weighted average feature set and the ring expansion area respectively allow for dimensionality reduction in the feature space, without losing relevant information by discarding some radiomics features that might result from using LASSO or other such feature selection techniques. A comparison of the methods and outcomes of our study with the two studies involving PSMA-PET/CT scans for primary prostate cancer mentioned above (labelled “Previous_1” [44] and “Previous_2” [45]) has been included in supplementary Table S5.

Our study is significant in the following aspects: (i) This is one of the first studies to explore the prognostic value of pre-treatment PSMA-PET, a relatively recent advancement in the care of prostate adenocarcinoma patients, for primary prostate cancer patients’ outcome prediction. Further, we have pre-registered CT scans along with the PET scans for this cohort, that gives us access to radiomics information from multi-modal imaging data. We observed that models built by combining the clinical variables and radiomics information from the regions of interest in both CT and PET scans gave us the highest prognostic performance, compared to using models built using clinical variables and radiomics information from the ROIs on CT or PET scans alone (supplementary Table S4). The model built using radiomics information from ROIs on the CT scans achieved comparable prognostic performance to the model built using radiomics information from the ROIs on the PET scans. Both models were inferior to the model using radiomics information from both CT and PET. This indicates that the CT and PET scans provide complementary information and thus combining them gives the highest prognostic performance. (ii) Our study uses a unique way (weighted average by volume) of combining the radiomics information from the primary tumor and node regions of interest, that improves the ability of the prognostic model in the prediction of MFS, compared to the model built using radiomics information from only the primary tumor. We further observed that the prognostic model built using only clinical variables (model 3) could not achieve a statistically significant separation in the KM curves stratified by five-year metastasis-free survival (log rank p value: 0.17) in the test data. The prognostic models built using clinical variables and radiomics information from primary tumor and nodes (model 1) resulted in a statistically significant separation in the KM curves stratified by five-year MFS (log rank p value: 0.042) in the test data (Fig. 3). (iii) We have also included radiomics information extracted from the 5 mm ring expansion area around the primary prostate tumor, that has further improved the performance of our prognostic models. These results are in accordance with observations from recent studies have explored the added value of features extracted from the periphery of the region of interest [46].

Notably, systemic treatment in this cohort was limited to LHRH agonists and bicalutamide, as next-generation ARPIs and upfront chemotherapy were not used. This reflects the treatment standards and healthcare policies during the accrual period, when the use of ARPIs in high-risk non-metastatic patients was not yet routine clinical practice. Their broader incorporation into guidelines such as NCCN and ESMO occurred around 2022, following publication of supporting trial data. Additionally, reimbursement constraints under the Turkish Social Security System restricted ARPI access to patients with metastatic castration-resistant disease. Consequently, our findings must be interpreted in the context of this treatment landscape. While this enhances the internal consistency of the cohort, it may also introduce a degree of selection bias, particularly in comparison to more contemporary treatment approaches involving intensified systemic therapy.

This study has some limitations. Mainly, the sample size of 134 patients is still relatively small, and there is a lack of external validation using independent, cross-institutional datasets. This limitation is primarily since PSMA-PET imaging has only been adopted in clinical practice in recent years, e.g. FDA approval in the US granted on December 1, 2020. Consequently, available PSMA-PET data on primary prostate cancer patients treated with radiotherapy, particularly those with long-term follow-up such as five-year MFS, remain very limited. Further, we initially included PSA relapse as a baseline variable based on the rationale that biochemical recurrence does not always coincide with radiographically evident metastasis. In some cases, patients may experience PSA relapse without developing detectable metastatic disease. Therefore, we hypothesized that a history of PSA relapse could serve as a potential predictor of future metastasis-free survival. However, we acknowledge that PSA relapse is a post-treatment outcome and its inclusion as a baseline covariate may introduce temporal bias. Despite these constraints, our study provides an important contribution by demonstrating the prognostic value of radiomic biomarkers derived from pre-treatment PSMA-PET/CT in predicting outcomes for patients with primary prostate cancer. In the future, more comprehensive validation of the imaging biomarkers will be warranted when more patient data becomes available at different institutions for this new imaging technology. In our future work, we also plan to investigate post-treatment follow-up PSMA-PET scans of the patients, in addition to the current pre-treatment scans, to assess the value of delta radiomics signatures in further improving the performance of the prognostic model in the prediction of metastasis-free survival. Further, recent studies have explored the value of information from dose distribution patterns (dosiomics) for risk assessment in lung cancer [47] and prognostic modeling in head and neck cancer patients [48]. In our future work, we also plan to assess the value of dose map information of the patients in improving the prognostic capability of the model in the prediction of metastasis-free survival in our prostate cancer cohort.

## Conclusion

This study addressed an important clinical gap by investigating the prognostic utility of radiomic biomarkers derived from pre-treatment PSMA-PET/CT scans, combined with clinical parameters, to predict metastasis-free survival in patients with primary prostate adenocarcinoma. Our findings underscore the potential of PSMA-PET/CT-based radiomics as a non-invasive, image-derived biomarker to support personalized treatment strategies and improve clinical decision-making in primary prostate cancer management.

## Supplementary Material

Supplementary material

## Figures and Tables

**Table 1:** Cox-regression analysis results for models 1, 2 and 3.

Model	Train (c-scores, 95 % CI)	Test (c-scores, 95 % CI)

Model 1 (primary + node + ring + clinical)	0.77 [0.72, 0.78]	0.69 [0.64, 0.70]
Model 2 (primary + ring + clinical)	0.72 [0.66, 0.73]	0.63 [0.58, 0.64]
Model 3 (clinical)	0.62 [0.57, 0.63]	0.54 [0.51, 0.56]

**Table 2:** Five-year MFS binary classification results for models 1, 2 and 3.

Model	Train [Sensitivity, Specificity, AUC]	Test [Sensitivity, Specificity, AUC]

Model 1 (primary + node + ring + clinical)	[83.6 %, 91.3 %, 0.88]	[76.3 %, 82.5 %, 0.81]
Model 2 (primary + ring + clinical)	[77.4 %, 85.1 %, 0.84]	[71.5 %, 78.2 %, 0.76]
Model 3 (Clinical)	[69.3 %, 78.2 %, 0.76]	[64.7 %, 72.6 %, 0.68]

**Table 3:** Assessing improvement in prognostic performance of the models (cox-regression analysis and five-year metastasis-free survival classification analysis) upon addition of ring radiomics to models 1 and 2.

Model	Train (c-scores, 95 % CI)	Test (c-scores, 95 % CI)	Test [Sensitivity, Specificity, AUC]

Model 1_with ring	0.77 [0.72, 0.78]	0.69 [0.64, 0.70]	[75.1 %, 81.4 %, 0.79]
Model1_without ring	0.75 [0.70,0.76]	0.67 [0.63,0.68]	[73.4 %, 78.5 %, 0.76]
Model 2_with ring	0.72 [0.66, 0.73]	0.63 [0.58, 0.64]	[70.2 %, 77.3 %, 0.74]
Model 2_without ring	0.70 [0.64,0.71]	0.61 [0.57,0.63]	[67.4 %, 74.6 %, 0.72]

## Appendix A. Supplementary data

Supplementary data to this article can be found online at https://doi.org/10.1016/j.radonc.2025.111119.
