# Supplementary material for: Prediction of metastasis-free survival in patients with localized prostate adenocarcinoma using primary tumor and lymph node radiomics from pre-treatment PSMA-PET/CT scans
Source: Radiother Oncol. Author manuscript; Available in PMC 2026 Feb 16. (PMC12908859; doi:10.1016/j.radonc.2025.111119)
Supplement: Supplementary material [file NIHMS2136846-supplement-Supplementary_material.docx]

**Prediction of metastasis-free survival in patients with localized prostate adenocarcinoma using primary tumor and lymph node radiomics from pre-treatment PSMA-PET/CT scans.**

Apurva Singh, PhD^1^, William Silva Mendes, MD^1^, Sang-Bo Oh, MD^1,4^, Ozan Cem Guler, MD^2^, Aysenur Elmali, MD^3^, Birhan Demirhan, MD^2^, Amit Sawant, PhD^1^, Phuoc Tran, MD, PhD^1*^, Cem Onal, MD^2,3*^, Lei Ren, PhD^1*^

^1^Department of Radiation Oncology, University of Maryland School of Medicine, Baltimore, MD, USA

^2^Baskent University Faculty of Medicine, Adana Dr Turgut Noyan Research and Treatment Center, Department of Radiation Oncology, Adana, Turkey

^3^Baskent University Faculty of Medicine, Department of Radiation Oncology, Ankara, Turkey

^4^Division of Medical Oncology and Hematology, Department of Internal Medicine, Pusan National University Yangsan Hospital, Pusan National University School of Medicine, Yangsan, Republic of Korea

**Table S1:** Patient demographic and clinical variables information

| **Variable** | **Category** | **Number of patients (n=134)** |
| --- | --- | --- |
| Age | Median, range | 69, [55, 87] |
| Gleason score | Low grade (≤ 7) | 62 (46.3%) |
|  | High grade (> 7) | 72 (53.7%) |

**Table S2**: A list of the radiomic features (107) extracted using PyRadiomics. The column headings indicate the family to which the list of features belongs to.

| **Shape** | **FirstOrder** | **GLCM** | **GLDM** | **GLRLM** | **GLSZM** | **NGTDM** |
| --- | --- | --- | --- | --- | --- | --- |
| Elongation | 10Percentile | Autocorrelation | Dependence  Entropy | GrayLevelNon  Uniformity | GrayLevelNonUniformity | Busyness |
| Flatness | 90Percentile | Cluster  Prominence | Dependence  NonUniformity | GrayLevelNon  Uniformity  Normalized | GrayLevelNonUniformity  Normalized | Coarseness |
| LeastAxis  Length | Energy | ClusterShade | Dependence  NonUniformity  Normalized | HighGrayLevel  RunEmphasis | GrayLevel  Variance | Complexity |
| MajorAxis  Length | Entropy | Cluster  Tendency | Dependence  Variance | LongRun  Emphasis | HighGrayLevelZone  Emphasis | Contrast |
| Maximum2D  Diameter  Column | InterQuartileRange | Contrast | HighGrayLevel  Emphasis | LongRunHigh  GrayLevel  Emphasis | LargeArea  Emphasis | Strength |
| Maximum2D  Diameter  Row | Kurtosis | Correlation | Large  Dependence  Emphasis | LongRunLow  GrayLevelRun  Emphasis | LargeArea  HighGrayLevelEmphasis |  |
| Maximum2D  Diameter  Slice | Maximum | Difference  Average | Large  Dependence  HighGrayLevel  Emphasis | LowGrayLevel  RunEmphasis | LargeAreaLowGrayLevel  Emphasis |  |
| Maximum3D  Diameter | Mean  Absolute  Deviation | Difference  Entropy | Large  Dependence  LowGrayLevel  Emphasis | RunEntropy | LowGrayLevelZone  Emphasis |  |
| Mesh  Volume | Mean | Difference  Variance | LowGrayLevel  Emphasis | RunLengthNon  Uniformity | SizeZoneNonUniformity |  |
| MinorAxis  length | Median | Id | Small  Dependence  Emphasis | RunLengthNon  Uniformity  Normalized | SizeZoneNonUniformity  Normalized |  |
| Sphericity | Minimum | Idm | Small  Dependence  HighGray  LevelEmphasis | RunPercentage | SmallArea  Emphasis |  |
| Surface  Area | Range | Idmn | Small  Dependence  LowGrayLevel  Emphasis | RunVariance | SmallArea  HighGrayLevelEmphasis |  |
| Surface  Volume  Ratio | RobustMean  Absolute  Deviation | Idn |  | ShortRun  Emphasis | SmallAreaLowGrayLevel  Emphasis |  |
| Voxel  Volume | RootMean  Squared | Imc1 |  | ShortRunHigh  GrayLevel  Emphasis | ZoneEntropy |  |
|  | Skewness | Imc2 |  | ShortRunLow  GrayLevel  Emphasis | Zone  Percentage |  |
|  | TotalEnergy | Inverse  Variance |  |  | ZoneVariance |  |
|  | Uniformity | JointAverage |  |  |  |  |
|  | Variance | JointEnergy |  |  |  |  |
|  |  | MCC |  |  |  |  |
|  |  | Maximum  Probability |  |  |  |  |
|  |  | SumAverage |  |  |  |  |
|  |  | SumEntropy |  |  |  |  |
|  |  | SumSquares |  |  |  |  |

**Table S3:** Comparing the prognostic performance of the models with data imbalance correction in model 1 (three PCs each from the weighted average of primary tumor and node radiomics (GTVp, GTVn) from CT and PET scans + first PC from ring region radiomics from CT and PET scans + clinical variables) and model 2 (three PCs each from the primary tumor radiomics (GTVp) from CT and PET scans + first PC from ring region radiomics from CT and PET scans + clinical variables).

| **Model** | **Train**  **(c-scores, 95% CI)** | **Test**  **(c-scores, 95% CI)** |
| --- | --- | --- |
| Model 1_withimbalancecorrection | 0.77 [0.72, 0.78] | 0.69 [0.64, 0.70] |
| Model1_withoutimbalancecorrection | 0.67 [0.62,0.68] | 0.60 [0.55,0.61] |
| Model 2_withimbalancecorrection | 0.72 [0.66, 0.73] | 0.63 [0.58, 0.64] |
| Model 2_withoutimbalancecorrection | 0.63 [0.58,0.64] | 0.54[0.50,0.56] |

**Table S4:** Comparing the prognostic performance of the models built by combining the clinical variables and radiomics information from the regions of interest in both CT and PET with the prognostic performance of the models built using clinical variables and radiomics information from the regions of interest on CT or PET scans alone.

| **Model** | **Train (c-scores, 95% CI)** | **Test (c-scores, 95% CI)** |
| --- | --- | --- |
| Model 1_CT+PT (primary+node+ring+clinical) | 0.77 [0.72, 0.78] | 0.69 [0.64, 0.70] |
| Model 1_CT  (primary+node+ring+clinical) | 0.74 [0.68, 0.75] | 0.65 [0.60, 0.66] |
| Model 1_PT  (primary+node+ring+clinical) | 0.73 [0.68, 0.74] | 0.64 [0.59, 0.65] |

**Table S5: Comparing previous studies involving PSMA PET/CT radiomics with the current study.**

| **Study** | **Number of patients** | **Outcome** | **Regions of interest** | **Construction of radiomics signature** |
| --- | --- | --- | --- | --- |
| Current | 134 | Metastasis-free survival | Primary tumor and pelvic nodes on PET/CT scans. | Weighted average method to combine radiomics information from primary tumor and nodes, radiomics principal component constructed from above averaged features forms the signature. |
| Previous_1 | 83 | Overall survival | Primary tumor and metastatic regions on PET/CT scans. | LASSO technique used to select most relevant radiomics features to form the signature. |
| Previous_ 2 | 125 | Discriminate between patients with prostate cancer and those with benign prostate disease | Primary tumor and benign prostate disease regions on PET/CT scans. | LASSO technique used to select most relevant radiomics features to form the signature. |

**Table S6: Taron-Ware test p values for Kaplan-Meier metastasis free survival curves of patients in the test data stratified by five-year MFS classification results.**

| **Model** | **Taron Ware test p values** |
| --- | --- |
| Model 1 (primary+node+ring+clinical) | 0.024 |
| Model 2  (primary+ring+clinical) | 0.041 |
| Model 3  (clinical) | 0.17 |

**Note 1:** **Sample size consideration- post hoc power analysis**

To estimate hazard ratio (HR) from c-index (Harrell’s 2001 EPV (events per variable) estimate:

C-index ≈ log(HR) / [log(HR) + log(2)].

Thus, for Model 1 test c-index (0.69), HR ~1.8

To estimate power, we use Schoenfeld approximation (1981):


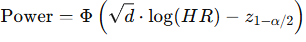


Assuming alpha (two-sided)- 0.05, d (number of events)- 24, log (HR), i.e., log (1.8) ~0.58,

- (CDF of normal distribution), from the standard normal tables ~0.82.

Thus, post hoc power is approximately 82%.
